# Role of asymptomatic and symptomatic humans as reservoirs of visceral leishmaniasis in a Mediterranean context

**DOI:** 10.1371/journal.pntd.0008253

**Published:** 2020-04-23

**Authors:** Ricardo Molina, Maribel Jiménez, Jesús García-Martínez, Juan Víctor San Martín, Eugenia Carrillo, Carmen Sánchez, Javier Moreno, Fabiana Alves, Jorge Alvar

**Affiliations:** 1 Laboratory of Medical Entomology, Centro Nacional de Microbiología, Instituto de Salud Carlos III, Majadahonda, Madrid, Spain; 2 WHO Collaborating Centre for Leishmaniasis, Centro Nacional de Microbiología, Instituto de Salud Carlos III, Majadahonda, Madrid, Spain; 3 Clinical Laboratory Service, Blood Bank, Hospital Universitario de Fuenlabrada, Fuenlabrada, Madrid, Spain; 4 Internal Medicine Service, Hospital Universitario de Fuenlabrada, Fuenlabrada, Madrid, Spain; 5 Drugs for Neglected Diseases Initiative, Geneva, Switzerland; Charité University Medicine Berlin, GERMANY

## Abstract

**Background:**

In the Mediterranean basin, *Leishmania infantum* is the causative agent of visceral leishmaniasis (VL), a zoonosis in which the dog is the primary domestic reservoir, although wildlife may have a leading role in the sylvatic cycle of the disease in some areas. Infections without disease are very frequent. There is limited information regarding the role that VL patients and asymptomatic infected individuals could be playing in the transmission of *L*. *infantum*. Xenodiagnosis of leishmaniasis has been used in this descriptive study to explore the role of symptomatic and asymptomatic infected individuals as reservoirs in a recent focus of leishmaniasis in southwestern Madrid, Spain.

**Methodology and main findings:**

Asymptomatic blood donors (n = 24), immunocompetent patients who were untreated (n = 12) or treated (n = 11) for visceral leishmaniasis (VL), and immunocompromised patients with VL (n = 3) were enrolled in the study. Their infectivity to *Phlebotomus perniciosus* was studied by indirect xenodiagnosis on peripheral blood samples. Quantitative polymerase chain reaction of blood samples from immunocompetent patients untreated for VL and immunocompromised untreated, treated and under secondary prophylaxis for VL was performed. Antibodies against *Leishmania* were studied by indirect fluorescent antibody and rK39-immunochromatographic tests. A lymphoproliferative assay with a soluble *Leishmania* antigen was used to screen for leishmaniasis infection in the healthy population. Sixty-two xenodiagnostic tests were carried out and 5,080 sand flies were dissected. Positive xenodiagnosis was recorded in four patients, with different sand fly infection rates: 1 immunosuppressed HIV / *L*. *infantum* coinfected asymptomatic patient, 1 immunosuppressed patient with multiple myeloma and symptomatic active VL, and 2 immunocompetent patients with untreated active VL. All blood donors were negative for both xenodiagnosis and conventional PCR.

**Conclusions / Significance:**

There is no consensus amongst authors on the definition of an ‘asymptomatic case’ nor on the tools for screening; we, therefore, have adopted one for the sake of clarity. Immunocompetent subjects, both infected asymptomatics and those treated for VL, are limited in number and appear to have no epidemiological relevance. The impact is limited for immunocompetent patients with untreated active VL, whilst immunosuppressed individuals undergoing immunosuppressive therapy and immunosuppressed individuals HIV / *L*. *infantum* coinfected were the most infectious towards sand flies. It is noteworthy that the HIV / *L*. *infantum* coinfected patient with asymptomatic leishmaniasis was easily infectious to sand flies for a long time, despite being under continuous prophylaxis for leishmaniasis. Accordingly, screening for latent *Leishmania* infection in HIV-infected patients is recommended in scenarios where transmission occurs. In addition, screening for VL in HIV-infected patients who have spent time in VL-endemic areas should also be implemented in non-endemic areas. More research is needed to better understand if some asymptomatic coinfected individuals contribute to transmission as ‘super-spreaders’.

## Author summary

Leishmaniasis is a set of diseases caused by *Leishmania* parasites and transmitted through the bites of infected phlebotomine sand fly females during blood ingestion. Visceral leishmaniasis (VL) is caused by *Leishmania infantum* in the Mediterranean region and dog is the primary domestic reservoir, although we can not ignore the role that wild reservoirs can have under certain conditions. As for the role of the human host in the transmission of the disease is not fully understood. The present work explores this important epidemiological aspect to try to clarify it. The study concludes that asymptomatic infected individuals and the immunocompetent patients treated for VL have no epidemiological impact in the transmission of *L*. *infantum*. As for the immunocompetent patients with untreated active VL its impact is limited, whilst immunosupressed patients are the most infectious towards sand flies. Thus, the screening for latent *Leishmania* infection in HIV-infected patients is recommended in scenarios where transmission occurs.

## Introduction

This study was conducted in an area in southwest Madrid called Fuenlabrada where an outbreak of human leishmaniasis occurred between 2010 and 2019 with more than 775 cases. The causative agent of visceral leishmaniasis (VL) in the Mediterranean basin is *Leishmania infantum* where the dog is the primary domestic reservoir. However, in the outbreak in question, it was proven that infected hares and rabbits were able to transmit the parasite to *Phlebotomus perniciosus*, the main vector in Western Europe [1, 2]. Moreover, the dog does not seem to have played a prominent role in the outbreak area [3] and there is scarce information on the role that humans may have in the transmission of the disease. This question is of special relevance since large numbers of subjects in endemic areas are infected with *Leishmania* spp. but do not develop any signs or symptoms of VL [4]. In an area of east Spain, about 50 people are infected for each symptomatic childhood case [5], although the role of asymptomatic carriers in transmission remains poorly understood. Studies on asymptomatic *L*. *infantum* infection in blood samples from donors in southeast Spain indicate that in rural donors PCR status was strongly related to the climate, altitude and soil type in the donor’s residence area [6]. The leishmanin skin test (LST) is the best tool for screening transmission, but in the absence of an LST produced under good clinical laboratory practices, WHO recommends–despite its operational limitations–the use of the cell lymphoproliferative assay (CPA) with peripheral blood mononuclear cells (PBMC) stimulated with soluble *Leishmania* antigen (SLA) when screening for leishmaniasis infection in a healthy population [7]. Moreover, the whole blood stimulation assay and cytokine analysis is a particularly useful method for detecting asymptomatic infections [8]. The ability of potential reservoir hosts to infect sand fly vectors is usually tested by xenodiagnosis [9] and this is, as far as we know, the best available tool to check the competence of a human to transmit *Leishmania* spp. to the sand fly vector. Both sand fly infection rate and parasite load are commonly used markers of infectiousness [10, 11].

As the role of the human host is not fully understood, the aim of the present work was to explore the role of asymptomatic and symptomatic infected individuals, both immunocompetent and immunosuppressed, in the transmission of *Leishmania* using the indirect xenodiagnosis test and quantitative polymerase chain reaction (qPCR), in the context of the outbreak area in Fuenlabrada, Madrid.

## Materials and methods

### Definition of asymptomatic infected individual

Someone with no clinical symptoms of leishmaniasis coming from or living in an endemic area who shows an immune response (either antibodies or a specific cellular response) against *Leishmania*, or who has parasites in the blood or any other tissue.

### Prepatent or incubation period

This is the time just before the patient suffers the first signs and / or symptoms of VL. This period is variable and lasts for several months, and is characterized by a solid serological response and / or detectable parasites, and the absence of a *Leishmania*-specific cell mediated response.

### Infectiousness to sand flies

The infectiousness to sand flies of symptomatic *Leishmania* infected patients was studied with heparinized peripheral blood (HPB) samples collected at the Internal Medicine Department of the Teaching Hospital of Fuenlabrada, Madrid. The infectivity of asymptomatic subjects was studied during screening of blood donors who attended the blood bank of the same hospital between 2015 and 2017. The donor population was constituted of healthy individuals older than 18 years with no previous history of clinical leishmaniasis, most of them living in *L*. *infantum*-endemic areas.

### Human subject groups

Patient groups with associated defining tests are summarized in Table 1.

**Table 1:** Associated defining tests for each patient group who underwent indirect xenodiagnostic.

Human subject groups	CPA-SLA	rK39- ICT	IFAT	PCR	qPCR
Active visceral leishmaniasis					
Treated visceral leishmaniasis					
Immunosupressed HIV / *L*. *infantum* coinfected patients					
Asymptomatic *Leishmania*-seropositive blood donors					
Asymptomatic blood donors					
Healthy blood donors with history of contact with *L*. *infantum*					
Healthy blood donors					

Gray colored cells indicate tests used in each patient group.

CPA, cell proliferation assay; ICT, immunochromatographic test; IFAT, anti-leishmanial antibodies by indirect fluorescent antibody test; IXD, indirect xenodiagnostic; SLA, soluble *Leishmania* antigen.

#### Active VL (n = 12)

Immunocompetent patients with fever for > 2 weeks, in combination with either enlargement of the spleen and / or liver, or weight loss. Diagnosis of leishmaniasis was confirmed by the rK39-immunochromatographic test (rK39-ICT) or by polymerase chain reaction (PCR).

#### Treated VL (n = 11)

Immunocompetent patients diagnosed with leishmaniasis by serology or PCR and cured after treatment with a total dose of 21 mg / kg liposomal amphotericin B (LAB) for 7 days who attended the hospital 3 months after treatment as outpatients for a medical check-up.

#### Immunosuppressed patients with leishmaniasis (n = 3)

- Patients undergoing immunosuppressive therapy (n = 1).
- Immunodepressed HIV / *L*. *infantum* coinfected patients (n = 2).

#### Asymptomatic *Leishmania*-seropositive blood donors (n = 2)

Subjects without clinical signs and symptoms of leishmaniasis and no prior history of the disease, with anti-leishmanial antibodies by indirect fluorescent antibody test (IFAT) and / or rK39-ICT, and a positive response to CPA-SLA.

#### Asymptomatic blood donors (n = 12)

CPA-SLA-positive subjects with no anti-leishmanial antibodies by IFAT and rK39-ICT, and negative PCR.

#### Healthy blood donors with a history of contact with *L*. *infantum* (n = 6)

CPA-SLA-negative subjects from an endemic area with a history of contact with the parasite months before (CPA-SLA-positive). All individuals with no anti-leishmanial antibodies by IFAT and rK39-ICT, and negative PCR.

#### Healthy donors (n = 4)

CPA-SLA-negative people with no history of VL that has not been in contact with the parasite. All subjects with no anti-leishmanial antibodies by IFAT and rK39-ICT, and negative PCR.

### CPA-SLA testing

The SLA extract was prepared from sonicated promastigotes of the stock (MCAN/ES/98/LLM-722), as described [12]. Heparinized PBMC were separated using a Ficoll-Hypaque gradient (Rafer, Spain), resuspended in complete RPMI supplemented with 10% foetal bovine serum, and cultured (in triplicate) at an initial concentration of 2 x 10^6^ cells / ml in 96-well plates with either complete RPMI (negative control) or SLA (10 μg / ml). All cultures were kept for 6 days at 37°C in a 5% CO_2_ atmosphere. The lymphoproliferative response of each subject was measured by bromodeoxyuridine incorporation using the Cell Proliferation Kit (GE Healthcare Life Sciences, UK), following the manufacturer’s instructions. Results were expressed in the form of a stimulation index (absorbance of SLA-cultivated cells / RPMI-cultivated cells). The cut-off for positive lympho-proliferation to SLA (CPA-SLA) was determined by calculating the area under the receiver operating characteristic curve (AUC) and the 95% confidence interval of the stimulation index (SI) value for 57 *Leishmania*-exposed but negative subjects and corresponds to a SI of ≥ 2.39.

### Detection of antibodies against *Leishmania*

#### IFAT

Antibody analyses of plasma samples were performed using 2 x 10^5^ *Leishmania* promastigotes (MHOM/FR/78/LEM-75) per well in PBS. The threshold titre for positivity was ≥ 1/80.

#### rK39-ICT

An rK39-based immunochromatographic rapid strip assay (Kalazar *Detect* InBios International, Inc.) was used following the manufacturer’s instructions.

### DNA extraction

DNA was extracted from 2 ml of subjects’ HPB by acid guanidinium-thiocyanate-phenol-chloroform extraction using the QIAamp DNA mini kit (QIAGEN) as described previously [13].

### PCR

Conventional *Leishmania*-nested PCR targeting the small subunit ribosomal RNA (SSU-rRNA) genes was performed with DNA isolated from the HPB of VL patients and donor participants, as explained previously [13].

### Quantitative PCR (qPCR)

qPCR was carried out as described previously [14]. Primers for the small subunit rRNA gene were used: 1000 nM of R223 and 500 nM of R333 (Sigma-Aldrich, USA). Total DNA was used as a template in touchdown qPCR reactions involving the LightCycler FastStart DNA Master SYBR Green I kit (Roche Applied Science, Switzerland).

### Sand flies

*Phlebotomus perniciosus* females from a colony established in 1987 in Madrid (Spain) were used. The colony was reared and maintained in an environmental chamber under controlled conditions of temperature (27°C ± 1°C), relative humidity (90–100%), and photoperiod (17:7 hours light-darkness) [15].

### Xenodiagnosis

HPB samples of all subjects stored at 4°C were sent to the laboratory, where indirect xenodiagnosis (IXD) was performed 24–48 h after blood collection except in two of them (see Table 2). *Phlebotomus perniciosus* females were used as described previously [16]. In brief, blood-feeding was carried out using 120–200 seven-day-old female *P*. *perniciosus* provided with HPB from each subject for 1 h in a feeding device. Surviving sand flies were dissected 4–5 days after feeding to determine the infection rate.

**Table 2:** Indirect xenodiagnosis of leishmaniasis performed with heparinized peripheral blood of immunocompetent patients with visceral leishmaniasis, untreated (N = 12, patient 1 to 12) and treated for visceral leishmaniasis who attended the hospital for check-up after treatment (N = 11, patient 13 to 23).

Patient	Age in years	Sampling date	rK39-ICT	PCR	qPCR Parasites/μl	Dissected / infected flies	IXD	Treated for VL
1^a^	72	07/07/2015	+	–	0	98/0	–	No
2	69	15/01/2016	+	+	152.6	106/0	–	No
3	33	01/04/2016	+	+	706	90/0	–	No
4	46	15/04/2016	+	–	0	100/0	–	No
5	42	28/04/2016	+	–	0	100/0	–	No
6	1.3	02/05/2016	+	–	0	66/0	–	No
7	88	04/05/2016	+	–	106	98/0	–	No
8^b^	45	09/05/2016	+	+	1050	73/4 (5.5%)	+	No
9	5	12/05/2016	+	+	27.3	62/0	–	No
10	54	11/07/2016	+	–	0	104/0	–	No
11	1.4	13/09/2016	+	+	99	104/1 (1%)	+	No
12^c^	41	22/11/2017	+	+	358	70/0	–	No
13	45	21/06/2016	+	–	ND	95/0	–	Yes
14	51	17/11/2015	–	–	ND	108/0	–	Yes
15	65	25/11/2015	+	–	ND	100/0	–	Yes
16	66	05/04/2016	+	–	ND	100/0	–	Yes
17	55	17/05/2016	+	–	ND	70/0	–	Yes
18	85	18/06/2016	+	–	ND	82/0	–	Yes
19	51	08/09/2016	+	–	ND	100/0	–	Yes
20	33	14/09/2016	+	–	ND	52/0	–	Yes
21	0.4	14/12/2016	+	–	ND	72/0	–	Yes
22	80	25/01/2017	–	+	ND	45/0	–	Yes
23	36	01/12/2017	+	–	ND	75/0	–	Yes

ICT, immunochromatographic test; IXD, indirect xenodiagnostic; ND, not done; VL, visceral leishmaniasis

^a^ Tracheal leishmaniasis.

^b^ IXD done 11 days after blood sampling.

^c^ IXD done 13 days after blood sampling.

### Statistical analysis

Data analysis was performed in GraphPad Prism 7.0.0 software. Univariate analyses used non-parametric tests as appropriate (significance *p* < 0.05). Wilcoxon matched-pairs signed-rank test was used to analyse the parasite load by qPCR and the sand fly infection rate in immunocompetent and immunocompromised patients. The correlation between parasite load and infection rate was determined using the Spearman’s test. The Mann-Whitney *U* test was performed to compare the infection rate and the parasite load in immunocompetent and immunocompromised patients.

### Ethical considerations

The project was reviewed and approved by the Human Research Ethics Committee of the Hospital of Fuenlabrada (APR12-65 and APR14-64). The procedures followed were in accordance with the ethical standards of the Committee on human experimentation and with the Helsinki Declaration (1964, amended most recently in 2008) of the World Medical Association. Informed written consent was obtained from the participants enrolled in the study. Sand flies used in xenodiagnosis were obtained from a colony held at Instituto de Salud Carlos III (ISCIII), Majadahonda, Madrid, Spain. Colony maintenance was performed according to the current guidelines and regulations of the Animal Protection Area of the ISCIII and in accordance with the terms of a regulated license (PROEX 200/15) of the Ministry of Environment of the Community of Madrid, Spain, in compliance with the applicable basic rules for the protection of animals used in experimentation.

## Results

From 16^th^ April 2015 to 1^st^ December 2017, 50 subjects were enrolled: 26 patients and 24 blood donors. Sixty-two xenodiagnoses were carried out and 5,080 sand flies were dissected. Four patients had positive xenodiagnosis: 1 immunosuppressed HIV / *L*. *infantum* coinfected patient with no symptoms of leishmaniasis, 1 immunosuppressed patient with multiple myeloma and active VL, and 2 immunocompetent patients with active VL. Blood samples from all donors were negative for both xenodiagnosis and PCR.

### Patients with active VL

Before receiving the treatment for leishmaniasis, 2 out of 12 (16.7%) immunocompetent patients (8 and 11) were positive by xenodiagnosis (Table 2), with sand fly infection rates of 5.5% and 1% respectively (mean infection rate, 3.3%). Of the 1,071 females dissected—fed with blood from all 12 patients—five (0.5%) were positive (Table 2). The blood samples of patients 2, 3, 7–9, 11 and 12 were positive (58.3%) by PCR and qPCR (Table 2). The rK39-ICT was positive in all patients. Patient 1 had been diagnosed with a tracheal mucocutaneous leishmaniasis.

Parasite loads and infection rates were significantly associated (*p* = 0.0156 by 2-tailed Wilcoxon matched-pairs signed rank test) but the correlation was not significant (*r* = 0.2673, *p* = 0.5714 by 2-tailed Spearman test).

### Patients treated for VL

The eleven immunocompetent patients cured after receiving treatment with a 21 mg / kg total dose of LAB for 7 days were negative by both xenodiagnosis and PCR when they attended the hospital for check-up, except for patient 22 who was positive by PCR (Table 2). A total of 899 female sand flies were dissected. The rK39-ICT performed the same day that the IXDs were carried out was positive in 9 out of 11 (81.8%) patients (13, 15–21, 23).

### Immunocompromised patients with leishmaniasis

Two of the three patients studied (66.7%) were positive by xenodiagnosis and 112 out of 1,213 dissected sand flies (9.2%) were positive (Table 3)

**Table 3:** Xenodiagnosis carried out with heparinized peripheral blood of immunosuppressed *Leishmania infantum* infected patients who attended the hospital for follow-up and prophylaxis or secondary treatment of leishmaniasis (N = 3).

Patient	Age in years	Sampling date	rK39 ICT	PCR	qPCR Parasites/μl	Dissected/ infected flies	IXD	CD4+ 10^6^/l	Viral load Copies/ml	Treatment/ secondary prophylaxis status	Clinical status
24	83	28/04/2016	+	+	4360	36/11 (30.6%)	+	ND	NA	Before LT	Immunosu- ppressed, multiple myeloma, SVL
	83	07/06/2016	+	–	0	86/0	–	ND	NA	Control Post-LT with LAB	
	83	17/10/2016	+	–	0	72/0	–	ND	NA		
25	40	01/07/2015	+	+	111.6	81/8 (9.9%)	+	122	<20	Before SPL	Immunosu-ppressed, HIV/ *L*. *infantum* coinfected, AL
	40	21/10/2015	+	+	148.8	71/18 (25.4%)	+	135	58	3 months after finish SPL with LAB	
	41	04/11/2015	+	+	608.4	105/51 (48.6%)	+	ND	107	5 months after finish SPL with LAB	
	41	09/12/2015	+	+	48.2	99/0	– ^a^	168	33	Just after 29 days of LT with M+F	
	41	17/02/2016	+	+	1104	103/20 (19.4%)	+	112	<20	2 months under SPL with MA+F	
	41	13/04/2016	+	+	824	112/9 (8.0%)	+	121	<20	4 months under SPL with MA+F	
	42	11/11/2016	+	+	658	56/2 (3.6%)	+	133	<20	1 year under SPL with MA+F	
26	50	24/04/2015	+	+	131.8	ND	ND	78	204	3 days after the start of LT with LAB	Immunosu-ppressed HIV/ *L*. *infantum* coinfected
	50	10/06/2015	+	–	0	108/0	–	121	85	Just after 29 days of LT with MA+F	
	50	21/10/2015	+	–	0	100/0	–	135	<20	14 weeks under LT with MA+F	
	51	27/11/2015	+	–	0	103/0	–	204	<20	2 weeks under SPL with LAB+F	
	51	15/06/2016	+	–	0	80/0	–	224	<20	6 months under SPL with MA+F	

AL, asymptomatic leishmaniasis; F, fluconazole; ICT, immunochromatographic test; IXD, indirect xenodiagnosis; LAB, liposomal amphotericin B; LT, leishmaniasis treatment; M, miltefosine; MA, meglumine antimoniate; NA, not applicable; ND, not done; SPL, secondary prophylaxis of leishmaniasis, SVL, symptomatic visceral leishmaniasis.

^a^ Haemolyzed blood.

#### Immunosuppressed patient with multiple myeloma lambda-IgA and active VL

Patient 24 was undergoing immunosuppressive therapy with lenalidomide + dexamethasone. The IXD performed before starting leishmaniasis treatment showed a sand fly infection rate of 30.6%. Leishmaniasis was then treated with LAB 3 mg / kg / day for 10 days. Two IXDs performed 5 and 22 weeks after treatment were negative (Table 3).

#### Immunosuppressed HIV / *L*. *infantum* coinfected patient with asymptomatic leishmaniasis

Patient 25 was undergoing highly active antiretroviral therapy (HAART) from 2011. He was diagnosed with leishmaniasis after a positive hepatic biopsy performed on April 23^rd^, 2012 and was repeatedly infective to sand flies by IXD from July 1^st^, 2015 to November 11^th^, 2016 without ever showing symptoms compatible with VL. Throughout this period, treatment and secondary prophylaxis for leishmaniasis were given to this patient (Table 4). Six of the seven (85.7%) IXD carried out were positive, the first one infected 9.9% of sand flies and the last one 3.6% (Table 3), with sand fly infection rates ranging from 3.6% to 48.6% (mean infection rate of 19.2%). The IXD performed on December 9^th^, 2015 was negative because the blood sample came haemolyzed to the laboratory, although *Leishmania* DNA was detected by PCR. All blood samples were positive by PCR and rK39-ICT.

**Table 4:** Therapeutic of visceral leishmaniasis and secondary prophylaxis administered to immunosuppressed patients during the study (N = 3).

Patient	Date of initiation of treatment or secondary prophylaxis	Drugs	Regimen
24	29/04/2016	LAB	3 mg/kg/day for 10 days
25	01/07/2014	LAB	3 mg/kg/month for 12 months
	11/11/2015	M + F	50 mg/8h/day + 800 mg/day for 29 days
	09/12/2015	MA + F	20 mg/kg/month + 800 mg/day for 11 months
26	21/04/2015	LAB	3 mg/kg/month for 2 months
	12/05/2015	MA + F	20 mg/kg/day + 200 mg/day for 29 days
	30/06/2015	MA + F	Booster dose of 20 mg/kg/every 21 days + 200 mg/day for 14 weeks
	02/11/2015	LAB + F	3 mg/kg/day + 800 mg/day for 5 days
	09/11/2015	LAB + F	3 mg/kg/week + 800 mg/day for 4 weeks
	11/12/2015	MA + F	Booster dose of 20 mg/kg/every 21 days + 800 mg/day for 6 months

F, fluconazole; LAB, liposomal amphotericin B, M: miltefosine; MA, meglumine antimoniate.

#### Immunosuppressed HIV / *L*. *infantum* coinfected patient with symptomatic VL

Patient 26 who was undergoing HAART was never infective to sand flies by xenodiagnosis. He was diagnosed with leishmaniasis after a positive bone marrow aspirate performed on March 14^th^, 2012 and 6 blood samples collected at different dates until April 24, 2015 were repeatedly positive by PCR to *L*. *infantum*. Treatment and secondary prophylaxis of leishmaniasis were given to this patient during the study (Table 4). The four IXD done from June 10^th^, 2015 to June 15^th^, 2016 were negative. All blood samples were positive to *L*. *infantum* by rK39-ICT (Table 3).

Parasite loads and sand fly infection rates were significantly associated (*p* = 0.0078 by 2-tailed Wilcoxon matched-pairs signed rank test) but the correlation was not significant (*r* = 0.4762, *p* = 0.2431 by 2-tailed Spearman test). The blood samples from immunosuppressed patients 24 and 25 were significantly more infective to sand flies than immunocompetent ones (*U* = 5.5, *p* = 0.0056 by 2-tailed Mann-Whitney test) while parasite load differences between immunosuppressed and immunocompetent patients were not significant (*U* = 19, *p* = 0.3357 by 2-tailed Mann-Whitney test).

### Asymptomatic *Leishmania*-seropositive blood donors

Anti-*Leishmania* IgG antibodies were detected by IFAT (1/160 titer) and rK39-ICT in donor 2 and by only rK39-ICT in donor 22. The IXD performed with the blood samples of both subjects were negative with a total of 182 females dissected (Table 5).

**Table 5:** Indirect xenodiagnosis performed with the heparinized peripheral blood samples of blood donors (N = 24).

Donor	Age in years	Sampling date	CPA- SLA	IFAT	rK39-ICT	PCR	Dissected / infected flies	IXD	Clinical status of subjects
1	48	16/04/2015	+	–	–	–	61/0	–	Asymptomatic
2	61	30/06/2015	+	+	+	–	89/0	–	Asymptomatic seropositive
3	43	01/07/2015	–	–	–	–	101/0	–	Healthy^a^
4	53	02/07/2015	–	–	–	–	71/0	–	Healthy^a^
5	42	07/07/2015	+	–	–	–	107/0	–	Asymptomatic
6	32	04/03/2016	–	–	–	–	58/0	–	Healthy
7	47	29/03/2016	+	–	–	–	82/0	–	Asymptomatic
8	30	30/03/2016	–	–	–	–	76/0	–	Healthy
9	54	30/03/2016	–	–	–	–	100/0	–	Healthy
10	60	19/04/2016	–	–	–	–	100/0	–	Healthy
11	44	11/05/2016	–	–	–	–	78/0	–	Healthy^a^
12	32	31/05/2016	–	–	–	–	77/0	–	Healthy^a^
13	29	04/10/2016	+	–	–	–	53/0	–	Asymptomatic
14	62	04/10/2016	+	–	–	–	66/0	–	Asymptomatic
15	55	05/10/2016	+	–	–	–	52/0	–	Asymptomatic
16	38	10/10/2016	+	–	–	–	96/0	–	Asymptomatic
17	29	17/10/2016	+	–	–	–	76/0	–	Asymptomatic
18	34	19/10/2016	+	–	–	–	84/0	–	Asymptomatic
19	34	24/10/2016	–	–	–	–	86/0	–	Healthy^a^
20	52	26/10/2016	+	–	–	–	59/0	–	Asymptomatic
21	34	09/11/2016	+	–	–	–	77/0	–	Asymptomatic
22	49	14/12/2016	+	–	+	–	93/0	–	Asymptomatic seropositive
23	45	15/02/2017	–	–	–	–	74/0	–	Healthy^a^
24	28	22/02/2017	+	–	–	–	81/0	–	Asymptomatic

CPA, cell proliferation assay; ICT, immunochromatographic test; IFAT, immunofluorescent antibody test; IXD, indirect xenodiagnosis; SLA, soluble *Leishmania* antigen.

^a^ Previous blood collection of these patients was CPA-SLA positive.

### Asymptomatic blood donors

All IXD performed with the blood of asymptomatic blood donors were also negative. A total of 894 females were dissected (Table 5).

### Healthy blood donors with a history of contact with *L*. *infantum*

All IXD were negative for these donors from the endemic area who had had a positive result to CPA-SLA in previous months. In total, 487 females were dissected (Table 5).

### Healthy blood donors

The IXD carried out on blood from donors who had never been in contact with the parasite were all negative. A total of 334 females were dissected (Table 5).

## Discussion

In recent years there has been an increasing interest in better understanding the role that asymptomatic infected individuals can play in the transmission of leishmaniasis; this is of particular relevance for elimination programs [17]. We present here a descriptive study on the infectivity of humans to sand flies using XD, based in a hospital in the Mediterranean context. Xenodiagnosis is the present gold standard for infectivity testing even though it is a complex technique that requires a high degree of specialization and the production of large numbers of sand flies in an insectary [18]. Although it is the best approach by now, xenodiagnosis may not provide a real measure of infectiousness because, above all, the model uses sand fly colonies kept in the insectary for years selecting the transmission ability. A frequent difficulty, even after ethical approval, is the refusal of a significant proportion of the individuals invited to be tested by direct xenodiagnosis (DXD), which is not the case if IXD is offered. IXD was the approach chosen in this study, despite its somewhat lower sensitivity as compared with DXD (personal unpublished data) which could be because the subclinical infection plays a positive role in the DXD.

The infectiousness of immunocompetent VL patients has already been reported in early papers [16, 19]. In our study, the blood of 7 out of 12 immunocompetent patients with active VL (58.3%) were positive by qPCR (mean parasite load of 356.9 parasites / μl), and only 2 of them (28.6%) were infectious to sand flies. As expected, since VL is a zoonosis in the Mediterranean context, this figure differs from that reported in Bangladesh with *Leishmania donovani*, which is typically an anthroponosis where the 66.7% of qPCR positive VL patients were positive by xenodiagnosis [10]. The mean proportion of patients with active VL who were infectious to sand flies in the present study was moderate, not very far from that recently reported in Brazil (16.7% vs 25%) using *Lutzomyia longipalpis*. Furthermore, the proportion of infected sand flies in our study was even lower than that recently reported in Brazil using DXD with *L*. *longipalpis* (0.9% vs 2.4%) [11]. Such differences can be attributed to the use of different xenodiagnostic methods with different sand fly species and may mean that blood and skin parasite loads are not strongly correlated in active VL although the sand fly infection rate reported in Brazil also was very low. The poor correlation between the parasite load in the blood and infectivity to sand flies observed in this group of patients suggests that their infectivity may be due to an additional source or parasites (i.e. skin) or to a random release of parasites. The parasite load in the blood of Mediterranean VL patients seems to be less infective when compared with VL patients from Bangladesh (16% vs 66.7%) [10]. On the other hand, it is interesting to note that the blood drawn from patient 8 was still infective after 11 days of storage at 4°C. Blood banks should take into consideration the possibility of analyzing samples preserved in this way. As for immunocompetent patients treated for VL and cured, it is noteworthy that none of them were infectious to sand flies, as had previously been reported elsewhere [11, 16].

**Fig 1:**
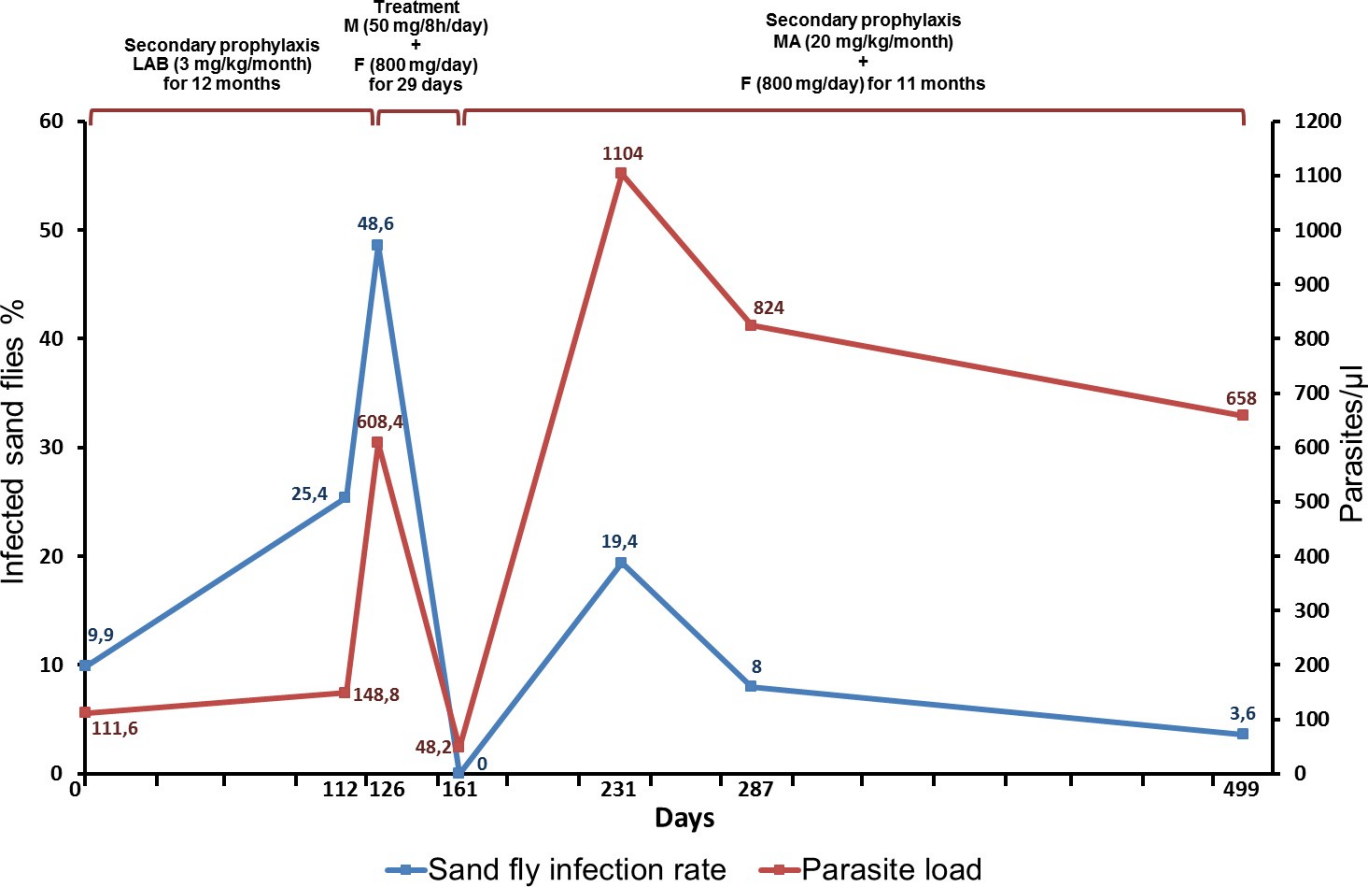
Therapeutic and secondary prophylactic regimens administered to HIV / *L*. *infantum* coinfected patient 25 after almost 17 months monitoring its infectivity towards sand flies by indirect xenodiagnosis. F, fluconazole; LAB, liposomal amphotericin B; MA, meglumine antimoniate; M, miltefosine.

The immunosuppressed patients with leishmaniasis yielded interesting results. Our results showed for the first time that a VL patient with multiple myeloma undergoing immunosuppressive therapy (patient 24) was infectious to sand flies before treatment with a significant infection rate, and that after being treated for leishmaniasis, the patient was no longer infective for at least 6 months. Results for the immunosuppressed HIV / *L*. *infantum* coinfected patients were contradictory. The first one (patient 25) was an asymptomatic case that continued to be infectious to sand flies with relatively high infection rates during the nearly 17 months of monitoring by IXD, despite having always been on treatment and secondary prophylaxis (Table 3). The patient took properly antiretroviral therapy and his HIV viral load was always under 100 copies / ml or even undetectable and his CD4+ level did not rise (Table 3). Incomplete immune reconstitution was probably due to the lack of control of the parasite, and at the same time the absence of immune recovery, which they contributed to prevent the parasite clearance. This may have been the reason why this patient was recurrently infectious to sand flies. A similar observation was previously made in a HIV-coinfected patient monitored for 5 years (Molina, unpublished). It is also remarkable that the level of infectivity towards the sand flies of this patient fell abruptly when was treated with miltefosine+fluconazole and that when the meglumine antimoniate+fluconazole-based secondary prophylaxis regimen was administered, his infectiousness was lower compared to the LAB-based regimen administered although such differences are not significant (*p* = 0.1143 by 2-tailed Mann-Whitney test). (Fig 1. Therapeutic and secondary prophylactic regimens administered to HIV / *L*. *infantum* coinfected patient 25 after almost 17 months monitoring its infectivity towards sand flies by indirect xenodiagnosis. F, fluconazole; LAB, liposomal amphotericin B; MA, meglumine antimoniate; M, miltefosine). The number of immunosuppressed individuals studied in the present work is scarce but previous works showed the infective capacity of coinfected patients on sand flies, which supports the results obtained in our study [16, 19, 20]. Although it has been proposed that in immunosuppressed *L*. *donovani*-infected patients the skin is a major source of parasites for the sand fly vector [21], the present study shows that in the case of HIV / *L*. *infantum* coinfection, venous blood also represents an important source of parasites for sand flies. The presence of *Leishmania* amastigotes in the peripheral blood of HIV-infected patients has been known about for years and an artificial cycle among intravenous drug users when sharing needles with infected blood has even been proposed [22]. The parasite load by qPCR in this patient’s blood samples does not correlate well with the sand fly infection rates obtained by xenodiagnosis, although there are clear differences when compared with those obtained from immunocompetent VL patients. It is well-known that coinfected patients are infective for sand flies [16, 20] and this has more recently been confirmed [11]. The skin parasite load seems a better predictor than blood in canine leishmaniasis and probably in PKDL. Patched presence of parasites in the skin of experimentally infected mice determines that the skin is not a homogenous system, opening a new dimension of the problem [21]. In addition, the proportion of VL patients harboring parasites in healthy skin is relatively low as recently published [23]. In this paper none of the 22 patients with VL (7 of them being HIV-coinfected) showed parasites through histopathology or immunochemistry, excluding one among the 7 coinfected. In previous studies, skin sections from 49 infected dogs were examined and parasites were found in 77.6%, whereas only 16.3% out of 43 patients with active VL showed parasites in healthy skin [24]. In the examples given, whether the blood of the VL patients was infective or not to the sand flies was not investigated but the fact that the presence of parasites in skin is relatively low indicates a moderate role in transmission. These studies carried out in Brazil, are closest to the situation in Spain (both regions *L*. *infantum* settings), rather than PKDL patients in Asia or Africa (*L*. *donovani*) or canine leishmaniasis (a totally different model). Whether such coinfected patients can play a role as ‘super-spreaders’ remains a hypothesis. The position and clustering of the cases in the Fuenlabrada outbreak speaks in favor of this theory [25] and, moreover, this could explain the long duration of the outbreak, despite the drastic measures taken to control the hares and rabbits. What is more, we must keep in mind that 17% of the leishmaniasis cases in the Fuenlabrada outbreak involved some degree of immunosuppression [26] and that two of the three immunosuppressed patients studied in the present work were infective for sand flies. In Morocco, *L*. *infantum* has been detected in the 3% of HIV-coinfected asymptomatic patients by direct microscopic examination of smears of buffy-coat cells stained with May-Grünwald Giemsa [27] and in Italy, *L*. *infantum* DNA is present in 16.5% of HIV-infected asymptomatic patients [28]. On the other hand, it is necessary to draw attention to the fact that these patients are not systemically ill and may remain untreated for long periods, in the meantime being potentially infectious to sand flies [29].

Although asymptomatic infection with the *Leishmania* parasite was recently reported in a large percentage of the population in Fuenlabrada by whole blood stimulation assay with interleukin-2 quantification [30], no CPA-SLA positive asymptomatic donor was able to infect sand flies, including the two donors with positive serology for *Leishmania*. These results agree with those obtained by direct xenodiagnosis in Brazil in which no sand fly was found to be infected by microscopy, although the detection of *L*. *infantum* DNA by PCR in some *L*. *longipalpis* females fed on a few asymptomatic subjects was reported [11, 18]. However, several studies indicate that in some cases *L*. *infantum* is prevalent among asymptomatic blood donors [31–33] circulating intermittently and at low density in the blood of asymptomatic carriers [34]. Asymptomatic leishmaniasis is defined as infection in the absence of symptoms, a very broad description that has usually been recognized by leishmanin skin test, serological tests or PCR. In our study, the CPA-SLA assay was first used to select asymptomatic individuals for xenodiagnosis among the donors recruited. The *in vitro* CPA involving SLA is a valuable method for detecting immune reactivity to the parasite but its presence in the blood donors of our study must have been transient, which is why the parasites were not detected by qPCR or xenodiagnosis [35]. In the absence of immunosuppression, a positive cellular test means a very low risk of subsequent VL [36]. Although asymptomatic immunocompetent individuals have aroused great interest in recent years, the results of our study do not suggest that they are playing an important role in the transmission of *L*. *infantum* in the Mediterranean region and that more emphasis should be given to the infectivity of HIV-VL coinfected patients. Even though asymptomatic, patient 25 was HIV coinfected, a condition that doesn’t not represent what happens in VL immunocompetent patients, it is well established that blood of immunosuppressed patients is more infective to sand flies than blood from immunocompetent subjects due to circulating parasites [16, 19, 20]. Additionally, blood banks must manage blood (including leukoreduction) from donors living in endemic areas of leishmaniasis with particular caution to prevent transmission of leishmaniasis by transfusion [32, 37].

## Conclusions

Our study ranks immunosuppressed VL patients at the top of the list of human hosts when it comes to the ease of transmitting *L*. *infantum* to the sand fly vector of leishmaniasis. At the other end of the scale, asymptomatic subjects and immunocompetent patients treated for leishmaniasis are at the bottom of the list because none of them were able to infect any sand fly. Immunocompetent patients with active VL are placed in an intermediate position since only a few were able to transmit the parasite to some sand flies. Consequently, special attention should be paid to immunosuppressed HIV / *L*. *infantum* coinfected patients and, within this group, to those who are asymptomatic since they can easily infect sand flies for long periods of time even whilst under periodic secondary prophylaxis for leishmaniasis. Therefore more research is needed to better understand if some asymptomatic coinfected individuals contribute to the transmission as true ‘super-spreaders’. It would also be interesting to extend the study of immunosuppressed individuals not coinfected with *Leishmania*. On the other hand it is very important to definitely clarify the real role of the blood and skin of immunocompetent and immunosuppressed individuals.

In any case, the definition of an ‘asymptomatic case’ should be revisited and a consensus achieved about how to express it more accurately, as this will allow the data obtained in different studies to be comparable. Even though screening for latent *Leishmania* infection in HIV-infected patients is not recommended [7] it would be very advisable in some scenarios such as the Fuenlabrada focus in Spain, as the detection of potential human ‘super-spreaders’ of leishmaniasis [6, 25] would allow the implementation of appropriate control measures to prevent them from making contact with sand fly vectors.

Finally, a ‘screen and protect’ strategy would be advisable. In addition to this, screening of HIV-infected patients who have spent sufficient time in VL-endemic areas for VL should be implemented in non-endemic areas [38]. The IXD certainly does not allow large-scale screening, so it remains a priority to find a good infectivity marker that is at least equivalent to this technique.
